# Patient Perceptions of Video Visits Using Veterans Affairs Telehealth Tablets: Survey Study

**DOI:** 10.2196/15682

**Published:** 2020-04-15

**Authors:** Cindie Slightam, Amy J Gregory, Jiaqi Hu, Josephine Jacobs, Tolessa Gurmessa, Rachel Kimerling, Daniel Blonigen, Donna M Zulman

**Affiliations:** 1 Center for Innovation to Implementation Veterans Affairs Palo Alto Health Care System Menlo Park, CA United States; 2 Division of Primary Care and Population Health Stanford University School of Medicine Stanford, CA United States; 3 Program Evaluation and Resource Center Veterans Health Administration Menlo Park, CA United States; 4 National Center for Post-Traumatic Stress Disorder Veterans Affairs Palo Alto Health Care System Menlo Park, CA United States

**Keywords:** veterans, telehealth, telemedicine, eHealth

## Abstract

**Background:**

Video-based health care can help address access gaps for patients and is rapidly being offered by health care organizations. However, patients who lack access to technology may be left behind in these initiatives. In 2016, the US Department of Veterans Affairs (VA) began distributing video-enabled tablets to provide video visits to veterans with health care access barriers.

**Objective:**

This study aimed to evaluate veterans’ experiences with VA-issued tablets and identify patient characteristics associated with preferences for video visits vs in-person care.

**Methods:**

A baseline survey was sent to the tablet recipients, and a follow-up survey was sent to the respondents 3 to 6 months later. Multivariate logistic regression was used to identify patient characteristics associated with preferences for care, and we examined qualitative themes around care preferences using standard content analysis methods for coding the data collected in the open-ended questions.

**Results:**

Patient-reported access barriers centered around transportation and health-related challenges, outside commitments, and feeling uncomfortable or uneasy at the VA. Satisfaction with the tablet program was high, and in the follow-up survey, approximately two-thirds of tablet recipients preferred care via a tablet (194/604, 32.1%) or expressed that video-based and in-person care were “about the same” (216/604, 35.7%), whereas one-third (192/604, 31.7%) indicated a preference for in-person care. Patients were significantly more likely to report a preference for video visits (vs a preference for in-person visits or rating them “about the same”) if they felt uncomfortable in a VA setting, reported a collaborative communication style with their doctor, had a substance use disorder diagnosis, or lived in a place with better broadband coverage. Patients were less likely to report a preference for video visits if they had more chronic conditions. Qualitative analyses identified four themes related to preferences for video-based care: perceived improvements in access to care, perceived differential quality of care, feasibility of obtaining necessary care, and technology-related challenges.

**Conclusions:**

Many recipients of VA-issued tablets report that video care is equivalent to or preferred to in-person care. Results may inform efforts to identify good candidates for virtual care and interventions to support individuals who experience technical challenges.

## Introduction

### Background

Health care technology is advancing at a rapid pace and with it the opportunities for health care organizations to engage with patients in new ways [1]. This growth includes the expansion of telehealth services and technologies and now encompasses Web-based care solutions that include patient portals with email communication, access to records, technology to track chronic conditions remotely, video visits, and other resources that can improve access to health care [1-3].

The expansion of these Web-based care services includes the use of video visits to treat patients via mobile apps or Web-based applications [2,3]. The 2017 American Hospital Association Annual Survey found that 66% of health systems have adopted video visits, with variation across academic (61%), rural (25%), and community (45%) hospitals [4]. Video visits can help address issues related to health care access [5-7], and evidence shows that they can be effective methods for delivering care, especially for disease management [8-11] and mental health care [12-15]. Patients often report positive feedback and the desire to continue participating in video visits, citing the convenience, cost and time savings, and the benefit of facilitating timely access to care [16-18]. Prior work has identified the benefits of providing mental health care using video visits, including reduced treatment drop out and improvements in patient and provider satisfaction [9,10,13,15]. Mental health patients may also experience increased connectedness and support, improved privacy, and reduced treatment stigma [12], while providers of patients with mental health and chronic conditions may benefit from visual access to the patient’s home environment and other contextual information [9,10,12,13]. Some barriers to implementing video visits still exist, including cost and liability, training and support, and providers' willingness to engage [19-22]. Prior research, for instance, has found that some mental health clinicians felt that video visits could disrupt the clinical workflow or could be perceived as impersonal by some patients [22]. Health care systems continue to address these barriers, including recent changes in reimbursement for video visits [23].

With the rapid growth of Web-based care technologies and video visits, there is an interest in understanding patient attitudes toward these services [24,25], including patient experiences with video visits [16,18,26] and drivers of video visit adoption [27-30]. Previous studies have explored patient characteristics (eg, gender, age, education, and rurality) associated with video visit experiences and found that patient perceptions may improve the acceptance of video encounters [26,31]. Prior experience with the internet and technology [27,31] and the presence of health information seeking and socially motivated personality traits are also potentially associated with a greater willingness to participate in video visits [28].

Despite the promise of in-home video care and acceptance by many patients, access is frequently limited to patients who have a suitable device and the capability of accessing the internet. Patients without technology may not have the opportunity to realize any benefits of Web-based care [32]. This disparity may be an especially important issue for veterans, many of whom experience financial challenges that limit access and use despite their interest and willingness to engage in health technology [24,25]. The US Department of Veterans Affairs (VA) has long supported the use of technology to improve access to care and was an early adopter of video teleconferencing [1,33]. However, until recently, video visits were limited to veterans who could travel to community-based outpatient clinics to connect with providers at other facilities. As the VA developed plans to roll out in-home video visits, there were concerns that the technology requirements would generate new disparities for the many veterans with financial challenges, limiting their technology access and use [23,24].

To address this issue, the VA’s Offices of Rural Health and Connected Care developed a pilot initiative to distribute video-enabled tablets to veterans who did not have the necessary technology and who had a geographic, clinical, or social barrier to in-person health care access. A previous evaluation of this program suggests that the tablets were largely used for mental health care and that as many as 20% of tablet recipients did not use their tablets [34,35].

### Objectives

To inform optimal tablet distribution and technical support, we evaluated patient experiences with the initiative to learn about the characteristics of patients who prefer video visits to in-person appointments. Our primary objectives were to (1) identify the primary health care access barriers among VA-issued tablet recipients, (2) examine patient experiences with tablets and any changes in perceived access to care, and (3) investigate the patient characteristics associated with preferences for video visits vs in-person care**.**

## Methods

### Distribution of Tablets Issued by the Department of Veterans Affairs

In 2016, the VA launched a pilot initiative to distribute video-enabled tablets with 4G wireless broadband or Wi-Fi connectivity to veterans with access barriers. Providers could refer patients to the program if they had a clinical need for services but experienced a barrier to accessing VA care in person and if they lacked a device or the necessary internet connectivity to engage in video appointments [34]. Eligible patients received a video-enabled tablet with built-in wireless connectivity and the option to connect peripheral devices (such as a blood pressure cuff or thermometer) if indicated by the provider. Tablets allowed access to VA-supported programs such as the patient portal for managing prescriptions and secure messaging (My Health*e*Vet), mobile apps, and videoconferencing software. Tablets could be used for a wide range of clinical services, and the specific services and scheduling procedures were determined by local facilities. Technical support was available and provided by local VA facility telehealth coordinators and the VA National Telehealth Technology Help Desk.

The implementation of the VA’s tablet distribution initiative has been described previously [34]. Briefly, over the 2-year pilot period, 5000 tablets were distributed to 6745 patients at 86 (out of 130) VA health care systems, spanning all 18 geographic regions of the VA’s health care network. Approximately half of the tablet recipients lived in rural areas and 75% had a mental illness diagnosis. Tablets were predominantly used for mental health care [35] but also for spinal cord injury care, primary care, palliative care, rehabilitation, and other services [34]. The high rates of tablet use for VA mental health services are consistent with the early adoption of telemental health care in the VA [36] and likely explain the high rates of mental health conditions among tablet recipients compared with the general VA population.

### Patient Survey

As part of the program’s evaluation, the VA tablet shipment facility (Denver Acquisitions and Logistics Center, DALC) distributed surveys with all tablet shipments between April 1 and September 30, 2017. The paper survey packets included an initial incentive (US $2 or four first class US stamps), and those who completed surveys received US $10 as a thank-you. Participants could opt out at any time by calling or sending in an opt-out card to the evaluation team. Survey recipient information was provided to the evaluation team by the DALC, and the information was merged with administrative data to complete follow-up. Nonresponders received up to two reminder postcards and two additional survey copies as well as up to two follow-up phone calls within 2 months of the survey mail-out. Among the 2120 recipients of the baseline survey, 1321 returned the survey to the evaluation team, a 62% response rate. Similar procedures were used to send a follow-up survey to baseline survey respondents who had valid contact information 3 to 6 months later (n=1298). A total of 36.04% (763/2120) recipients completed both the baseline and follow-up survey and were included in these analyses. The survey-based evaluation of this quality improvement initiative was reviewed and designated as nonresearch by the supporting VA program office, local institutional review board, and VA Research Administration.

### Survey Measures

#### Baseline Characteristics

Patient-reported barriers to accessing health care were assessed using a list of eight potential barriers generated through a literature review and expert recommendations; the 4-point response options ranged from “not a problem” to “big problem” (see survey in Multimedia Appendix 1 [37-40]). The baseline survey also queried patients about characteristics that might influence engagement in video visits, based on a literature review and expert guidance, including the following: demographics, current experience and reliance on VA services (in-person and video), and experience using technology for health-related purposes, via general resources (eg, internet or social media and apps) and VA resources (eg, the VA’s patient portal for managing prescriptions and secure messaging, My Health*e*Vet, and telehealth remote monitoring for chronic conditions) [41]. Health literacy was measured using one item (“How confident are you filling out medical forms by yourself?”) developed and validated by Chew et al [42]. The collaborative communication style was assessed using questions from a current VA project that is developing a measure to assess veteran health care engagement (“When I see my provider I bring a list of questions or concerns I want to talk about”; “I can make sure my concerns are fully addressed before my appointment ends”; response options ranged from “Not true” to “Mostly true”) [43]. VA reliance was measured with questions in which patients indicated where they receive the majority of their primary, mental health, emergency, and hospital care (response options included “Mostly at the VA,” “Mostly outside VA,” “Half in VA, half outside VA,” and “Nowhere”). The survey also assessed demographics including education, household income, and level of economic hardship (“My household can make ends meet”) [44].

#### Outcome Measures

The follow-up survey evaluated overall experience and attitudes with the tablet, including improvements in access, perception of the video appointment, and feedback about technical aspects of the tablet technology. Patients were asked about their preference for future encounters (response options: video, in person, or “about the same”) and were provided space to describe the reasons for selecting their preference.

Both baseline and follow-up surveys included measures related to satisfaction with VA care overall, as well as primary care and mental health care evaluated on a 10-point scale (1=very dissatisfied and 10=very satisfied) adapted from the 2013 Customer Satisfaction Index [45].

#### Additional Data Sources

Administrative data were collected from the VA Corporate Data Warehouse [46] and included age, gender, race, ethnicity, marital status, and the number of chronic condition diagnoses in the year before receiving the tablet (defined using International Statistical Classification of Disease (ICD)-9 and ICD-10 codes) [47]. Distance from primary VA facility, patient’s zip code, and rural or urban designation were obtained from the VA’s Planning System Support Group. Rurality is defined based on the Rural Urban Commuting Area categories developed by the Department of Agriculture and Health and Human Services’ Health Resources and Services Administration [48]. The contractor (Iron Bow Technologies, Inc) of the VA tablet and the wireless internet provider shared information about the percentage of residents with 4G coverage per zip code.

### Quantitative Data Analysis

Following data cleaning, the missing data rate was less than 5% for all variables included in the analyses; no adjustments were made for the missing data. To improve the match of the survey respondents (n=764) to the demographics of the entire tablet cohort (n=5981), we performed poststratification survey weighting based on age, rurality, and presence of a mental health condition. To model the covariance among the eight health care access barriers in the survey, we performed exploratory factor analyses, with a predefined factor cutoff of 0.55 [49]. For regressions, Likert scale variables were dichotomized to group together “mostly true and very true,” “strongly agree and agree,” and “neutral, disagree, and not true” as the reference variable. Other continuous variables were dichotomized or grouped into categories (eg, distance and age). The total number of conditions was a continuous variable. All quantitative analyses were performed using Stata 15.0 (StataCorp, LLC, Texas, USA).

The primary outcome for the analyses was a patient-reported preference for video visits (vs a preference for in-person care or rating in-person and video-based care as equivalent). We used logistic regression models to identify patient characteristics associated with a preference for video visits. Three sensitivity analyses were conducted, the first combined video visits and equivalent ratings (vs in-person) and the following two analyses did not combine the three-item survey response options for care preferences: a multinomial logistic regression (video, in person [base], and “about the same”), and an ordered logistic regression ranking the response options (in person, “about the same”, and video).

An additional goal was to describe patient satisfaction with the VA tablet program and to understand changes in satisfaction with VA services by conducting paired *t*-tests among the baseline and follow-up survey respondents.

### Qualitative Data Analysis

The follow-up survey included one open-ended question after patients indicated their preference for future care: “Please explain the preference indicated for receiving VA care.” We analyzed qualitative responses to identify additional information from the patient perspective that could be used to identify patients who may prefer to use video visits. After removing blank responses, “not applicable” responses, and responses that did not answer the question, we analyzed qualitative data from 638 survey respondents using standard content analysis methods for coding open-ended textual data [50]. An initial codebook was developed using the eight barriers listed in the baseline patient survey and revised with additional codes after an independent review of the first 300 responses by 2 coders (CS and AG). The 2 coders independently coded the responses, and discrepancies were resolved by consensus. The codes were grouped into mutually exclusive themes that highlighted each patient’s care preferences. We identified exemplary quotes to demonstrate and highlight examples for each of the themes.

## Results

### Survey Respondents

**Figure 1:**
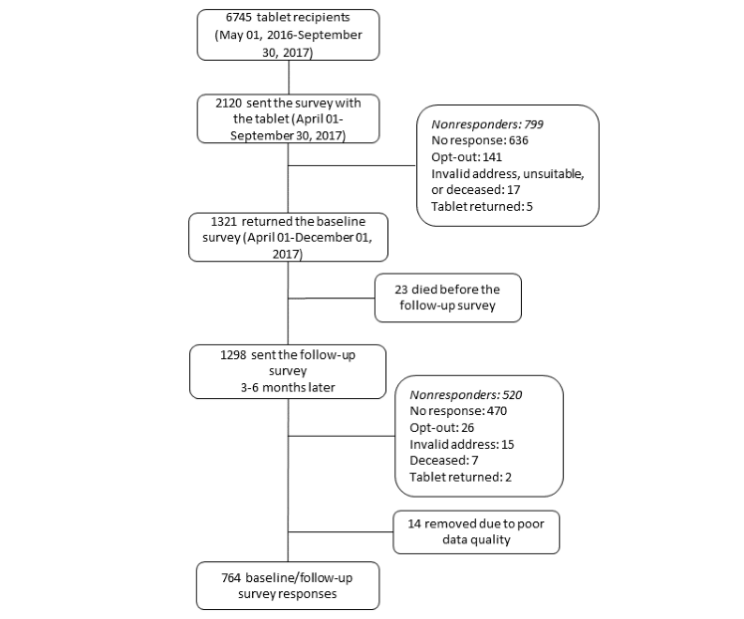
Survey response flow chart.

A full response breakdown is listed in Figure 1. The survey sample (n=764) was largely representative of the overall tablet cohort (n=5981), but survey respondents were more likely to be young (<65 years) to be non-Hispanic blacks, to live in an urban area, to have a greater number of mental health conditions, and to use the tablet at 6 months compared with the tablet cohort. A comparison of the survey cohort pre-and postweighting is available in Multimedia Appendix 2. Table 1 compares the unweighted characteristics of the baseline and follow-up survey respondents with baseline survey respondents only; the follow-up survey respondents were more likely to be older, to live in a rural area, and to use the tablet at 6 months compared with baseline responders. The mean (SE) age of the survey respondents was 56 (0.20) years, where 81.7% (624/764) of the respondents were men and 54.6% (412/754) lived in a rural location. The common chronic conditions are listed in Table 2, including hypertension (394/764, 51.6%), depression (389/764, 50.9%), and post-traumatic stress disorder (348/764, 45.5%). These condition rates are similar to the overall tablet cohort, and a comparison of the condition counts can be seen in Multimedia Appendix 2 [33]. The patient-reported access barriers are listed in Table 3; the big and small barriers were combined for this analysis owing to small cell sizes in a few barrier categories. The most common barriers were travel time (503/757, 66.4%), travel cost (416/753, 55.2%), health conditions (405/754, 53.7%), bad weather (426/754, 56.5%) and feeling uncomfortable or uneasy at the VA (248/753, 32.9%). The factor analysis created three categories of barriers from the original list: transportation, outside commitments (eg, work, school, and caregiving responsibilities), and a single barrier related to feeling uncomfortable or uneasy at the VA (Table 3).

**Table 1:** Demographics of the survey cohort.

Demographics^a^		Baseline and follow-up survey respondents (n=764)			Baseline survey respondents only (n=530)			*P* value	
		n (%)	Mean (SD)	Median (IQR)	n (%)	Mean (SD)	Median (IQR)		
**Age (years)^b^**			**58.6 (14.5)**			**54.7 (17.1)**			***<.001*^c^**
	18-44	137 (18.5)	–^d^	-	167 (32.4)	–	-		–
	45-64	306 (41.2)	–	-	173 (33.5)	–	-		–
	65-101	299 (40.3)	–	-	176 (34.1)	–	-		–
Male (%)		624 (81.7)	–	-	442 (83.4)	–	-		.42
**Marital status^b^**									
	Married	450 (58.9)	-	-	-	-	-		-
	Divorced or never married	287 (37.6)	-	-	-	-	-		-
	Widowed	17 (2.2)	-	-	-	-	-		-
**Race^b^**								**.17**	
	White or white non-Hispanic	598 (80.4)	-	-	388 (74.6)	-	-		–
	Black or African American	90 (12.1)	-	-	83 (16.0)	-	-		–
	American Indian, Native Hawaiian, or other	18 (2.4)	-	-	14 (2.7)	-	-		–
	Asian	4 (0.5)	-	-	5 (1.0)	-	-		–
	Unknown or decline	34 (4.6)	-	-	30 (5.8)	-	-		–
**Ethnicity^b^**								**.15**	
	Hispanic or Latino	32 (4.2)	-	-	35 (6.6)	-	-		–
	Non-Hispanic/Latino	704 (92.6)	-	-	475 (90.0)	-	-		–
	Unknown or decline	24 (3.2)	-	-	18 (3.4)	-	-		–
**Rurality^b,e^**								***<.01***	
	Urban	342 (45.4)	-	-	277 (53.2)	-	-		–
	Rural or highly rural	412 (54.6)	-	-	244 (46.8)	-	-		–
**Education^b,f^**								**.75**	
	Attended or graduated high school or general educational development	227 (29.8)	-	-	157 (30.0)	-	-		–
	Some college or 2-year degree	343 (45.1)	-	-	244 (46.7)	-	-		–
	4-year college graduate or more	191 (25.1)	-	-	122 (23.3)	-	-		–
**Income^b,f^ (US $ per year)**								**.32**	
	<25,000	249 (33.6)	-	-	186 (36.0)	-	-		–
	25,001-50,000	324 (43.7)	-	-	231 (44.7)	-	-		–
	>50,000	169 (22.8)	-	-	100 (19.3)	-	-		–
**Economic hardship^b,f^**								**.53**	
	Great difficulty and difficulty	243 (32.1)	-	-	172 (32.7)	-	-		–
	Some difficulty	271 (35.8)	-	-	203 (38.6)	-	-		–
	Rather easily	149 (19.7)	-	-	97 (18.4)	-	-		–
	Easily or very easily	94 (12.4)	-	-	54 (10.3)	-	-		–
Driving distance to primary VA^g^ facility (miles)		756 (98.9)	22.9 (22.9)	16 (7-32)	524 (98.9)	21.3 (22.5)	14 (6-30)		.21
Health literacy (out of 4)^f^		755 (98.8)	2.6 (1.3)	3 (2-4)	520 (98.1)	2.5 (1.3)	3 (2-4)		.18
Technology use pretablet^f^ (out of 8)		761 (99.6)	2.6 (1.9)	2 (1-4)	529 (99.8)	2.5 (1.9)	2 (1-4)		.29
Percentage of 4G internet coverage per zip code		760 (99.5)	96.5 (10.5)	99.99 (99.1-100)	527 (99.4)	97.4 (9.6)	99.99 (99.4-100)		.16
Number of tablet encounters at 6 months		592 (77.5)	5.7 (5.2)	4 (2-7)	363 (68.5)	4.4 (4.5)	3 (1-6)		*<.001*
Number of mental health tablet encounters at 6 months		383 (50.1)	5.7 (5.0)	4 (2-8)	246 (46.4)	4.7 (4.7)	3 (1-6)		*<.01*

^a^The results of poststratification weighing available in Multimedia Appendix 2 shows changes when weighted on age, rurality, and mental health conditions.

^b^The denominator for the proportions calculated is the total number of individuals with available data

^c^*P* values in italics are statistically significant.

^d^Not applicable.

^e^Rurality provided by the VA’s Planning Systems Support Group, which categorizes rural and urban status based on the Rural Urban Commuting Area categories developed by the Department of Agriculture and Health and Human Services’ Health Resources and Services Administration [48].

^f^Indicates a survey measure.

^g^VA: Department of Veterans Affairs.

**Table 2:** Chronic conditions among baseline and follow-up survey respondents.

Chronic conditions				Values				
				n (%)^a^		Mean (SD)		Median (IQR)
**Number of chronic conditions**						**4.1 (2.3)**		**4 (2-5)**
	0-3		297 (38.9)		–		–	
	4-6		339 (44.4)		–		–	
	7-14		128 (16.8)		–		–	
**Conditions^b^**								
	Acid-related diseases		181 (23.7)		-^c^		-	
	Alzheimer or Dementia		19 (2.5)		-		-	
	Arthritis		153 (20)		-		-	
	Asthma		43 (5.6)		-		-	
	Cancer		85 (11.1)		-		-	
	Chronic obstructive pulmonary disease		121 (15.8)		-		-	
	Diabetes		195 (25.5)		-		-	
	Heart failure		61 (8.0)		-		-	
	HIV or AIDS		4 (0.5)		-		-	
	Headache		97 (12.7)		-		-	
	Hepatitis C		30 (3.9)		-		-	
	Hypertension		394 (51.6)		-		-	
	Ischemic heart disease		114 (14.9)		-		-	
	Low back pain		279 (36.5)		-		-	
	Multiple sclerosis		17 (2.2)		-		-	
	Parkinson disease		25 (3.3)		-		-	
	Peripheral vascular disease		50 (6.5)		-		-	
	Prostatic hyperplasia		74 (9.7)		-		-	
	Renal disease		54 (7.1)		-		-	
	Spinal cord injury		62 (8.1)		-		-	
	Stroke		42 (5.5)		-		-	
	Traumatic brain injury		34 (4.5)		-		-	
**Mental health conditions**								
	Any mental health condition		561 (73.1)		-		-	
	Number of mental health conditions		-		1.3 (1.1)		1 (0-2)	
	**Substance use disorder^d^**		**121 (15.9)**		**-**		**-**	
		Alcohol abuse or dependence		90 (11.8)		-		-
		Drug use or dependence		63 (8.2)		-		-
	Schizophrenia		21 (2.7)		-		-	
	Bipolar disorder		50 (6.5)		-		-	
	Depression		389 (50.9)		-		-	
	Post-traumatic stress disorder		348 (45.5)		-		-	

^a^n (%) represents the unadjusted number of survey respondents; weighted differences are available in Multimedia Appendix 2.

^b^Conditions have been adapted from a list developed by the VA Health Economics Research Center [47].

^c^Not applicable.

^d^Substance use includes alcohol and any drug abuse or dependence diagnosis; individual and combined rates are shown.

**Table 3:** Self-reported health care access barriers and factor analysis.

Self-reported health care access barriers^a^	Big or small problem, n (%)	Not a problem or don’t know, n (%)	Factor 1	Factor 2	Uniqueness	Factor category
Travel time to the VA^b^ (n=757)	503 (66.4)	254 (33.6)	0.68	0.24	0.48	Transportation
Difficulty getting transportation to the VA (n=753)	270 (35.9)	483 (64.1)	0.71	0.01	0.49	Transportation
Cost of traveling to the VA (n=753)	416 (55.2)	337 (44.8)	0.60	0.30	0.55	Transportation
Health conditions make it challenging for you to get to the VA (n=754)	405 (53.7)	349 (46.3)	0.67	−0.06	0.55	Transportation
Bad weather conditions (n=754)	426 (56.5)	328 (43.5)	0.58	−0.23	0.61	Transportation
Work or school make it difficult for you to get the health care you need (n=734)	182 (24.8)	552 (75.2)	−0.07	0.84	0.28	Commitments
Family or caregiving responsibilities make it difficult for you to get the health care you need (n=753)	183 (24.3)	570 (75.7)	0.36	0.56	0.56	Commitments
Feeling uncomfortable or uneasy at the VA (n=753)	248 (32.9)	505 (67.1)	0.30	0.45	0.70	Uncomfortable or uneasy

^a^The predefined factor cut-off of .55 was used to group access barriers into categories.

^b^VA: Department of Veterans Affairs.

### Satisfaction With Tablets and the Department of Veterans Affairs Health Care

**Figure 2:**
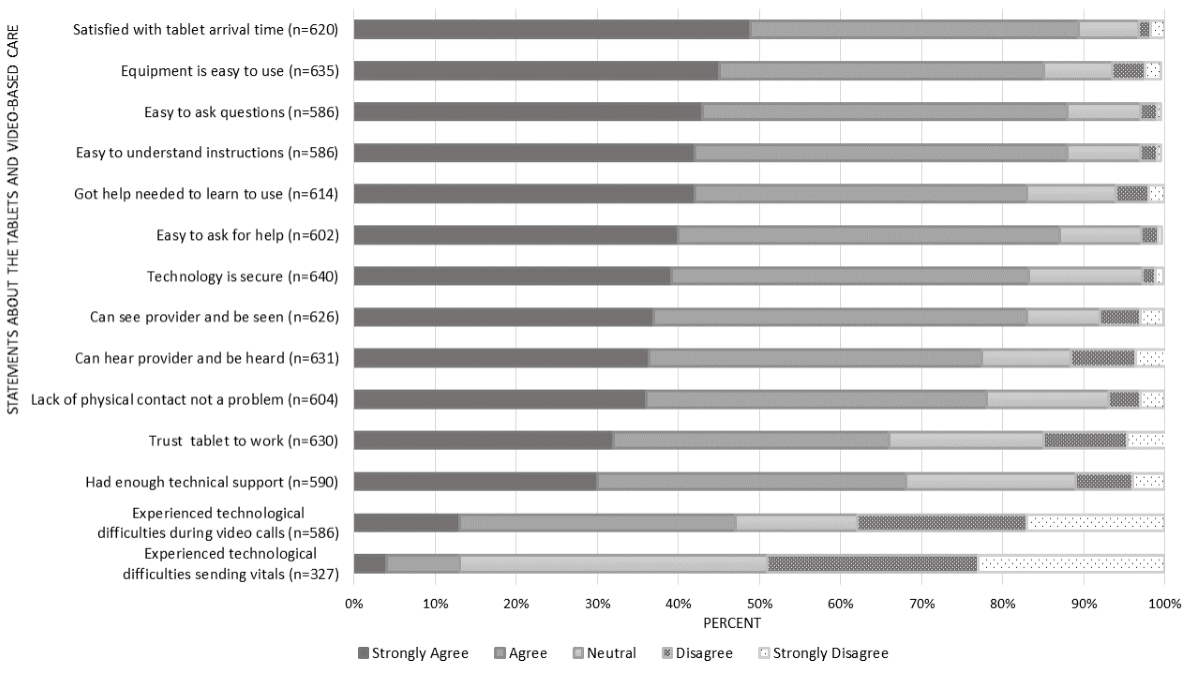
Patient-reported experiences using the tablets provided by the Department of Veteran Affairs.

Respondents indicated high levels of satisfaction with their VA health care. Between baseline and follow-up surveys, there were statistically significant increases in patient satisfaction regarding their overall VA care (from mean 7.4, SE 0.10 to mean 7.9, SE 0.08; *P*<.001; n=706), as well as primary care (from mean 7.4, SE 0.1 to mean 7.7, SE 0.1; *P*<.001; n=667) and mental health care (from mean 7.5, SE 0.1 to mean 8.2, SE 0.1; *P*<.001; n=570). In the follow-up survey, 86.0% (523/608) of the respondents indicated that they would recommend video care to others (agree or strongly agree). Satisfaction ratings for the quality of the technology and technical assistance were also high: 86.1% (547/635) agreed or strongly agreed with statements regarding the ease of using the equipment and receiving the help needed to learn the technology (83.5%, 513/614) and that it was easy to ask questions (88.4%, 518/586), ask for help (87.2%, 525/602), and understand instructions (87.4%, 512/586). Satisfaction with the video visits was also high: 84.1% (493/586) agreed or strongly agreed that their provider addressed their concerns during the video visit, 78.1% (472/604) agreed or strongly agreed that the lack of contact was not a problem, and 83.4% (534/640) agreed or strongly agreed that the technology was secure. A breakdown of these results is shown in Figure 2.

### Preferences for Video Visits Versus In-Person Care

In the follow-up survey, 32.1% (194/604) of tablet recipients indicated that they would prefer to conduct their future VA appointments by video, 31.8% (192/604) indicated that they would prefer these visits in person, and 35.7% (216/604) indicated their preference was “about the same”. In the multivariate regression analyses, patients were more likely to report a preference for video-based care (vs in person or “about the same”) if they reported the barrier of feeling uncomfortable or uneasy in the VA setting (adjusted odds ratio [AOR] 2.22, 95% CI 0.88-2.26; *P*<.001), if they indicated mostly/very true to the statement “I can make sure my concerns are fully addressed before my appointment ends” (AOR 1.59, 95% CI 1.02-2.47; *P*=.04), or if they had a substance use disorder in the year before receiving the tablet (AOR 1.91, 95% CI 1.12-3.26; *P*=.02). Patients were less likely to prefer video-based care if they had a greater number of chronic conditions (AOR 0.88, 95% CI 0.78-0.99; *P*=.03). There were trends suggesting that patients were also more likely to prefer video-based care if they had less than college education (AOR 1.52, 95% CI 0.96-2.40; *P*=.08) and if they indicated mostly/very true to the question “When I see my doctor, I bring a list of questions or concerns I want to talk about” (AOR 1.49, 95% CI 0.99-2.26; *P*=.06). The full regression results are listed in Table 4.

**Table 4:** Characteristics associated with the preference for video visits (N=558).

Preference for video appointments		Adjusted odds ratio^a^ (95% CI)		*P* value	
VA^b^ technology use^c^		1.41 (0.88-2.26)		.16	
Other technology use^c^		0.92 (0.60-1.41)		.70	
Reliance on VA: medical care^d^		0.93 (0.58-1.48)		.76	
Reliance on VA: mental health care^d^		0.70 (0.43-1.14)		.15	
**Driving distance to primary VA facility (miles; reference: <15 miles)**					
	16-40		1.24 (0.81-1.89)		.32
	>40		1.58 (0.89-2.79)		.12
Access barriers: transportation or travel^e^		1.44 (0.75-2.75)		.27	
Access barriers: commitments^e^		1.10 (0.72-1.67)		.66	
Access barriers: uncomfortable or uneasy^e^		2.22 (1.43-3.44)		*<.001*	
Gender (reference: male)		0.93 (0.56-1.54)		.78	
**Age (years; reference: 18-44 years)**					
	45-64		1.01 (0.62-1.66)		.96
	65-101		0.72 (0.38-1.37)		.32
Married^a^		1.35 (0.90-2.02)		.14	
Verizon coverage (reference: less than 95% coverage)		1.60 (0.84-3.06)		.15	
Economic hardship (great and some difficulty making ends meet vs all else)		1.43 (0.94-2.19)		.10	
**Education (reference: some college or more)**					
	High school graduate or GED^f^		1.52 (0.96-2.40)		.08
When I see my provider, I bring a list of questions or concerns I want to talk about^d^		1.49 (0.99-2.26)		.06	
I can make sure my concerns are fully addressed before my appointment ends^d^		1.59 (1.02-2.47)		*.04*	
Health literacy (quite and extremely vs all else)		1.08 (0.71-1.64)		.73	
Total number of conditions (continuous)		0.88 (0.78-0.99)		*.03*	
Substance use diagnosis^c^		1.91 (1.12-3.26)		*.02*	
Depression^c^		0.97 (0.63-1.49)		.89	
Post-traumatic stress disorder^c^		1.32 (0.87-2.02)		.19	
Schizophrenia or bipolar^c^		1.45 (0.71-2.95)		.31	

^a^Multivariate logistic regression comparing characteristics of patients who reported a preference for video visits with those who reported a preference for in-person care or reported a preference for video visits and in-person care “about the same” (reference group). Italicized *P* value indicate significance at *P*<.05.

^b^VA: Department of Veterans Affairs.

^c^Any or yes vs none.

^d^Mostly true and true vs all else.

^e^Big or small problem vs not a problem and don’t know.

^f^GED: general educational development.

We conducted several sensitivity analyses to understand the nuances among the survey question response options. The first analysis grouped patient-reported preferences for video visits with rating in-person and video-based care as equivalent and compared this with a preference for in-person care. In this model, patients were more likely to prefer video visits or report that they were equivalent to in-person care if they lived within a driving distance of 16 to 40 miles (AOR 1.65, 95% CI 1.09-2.51; *P*=.02) and were less likely to report these preferences if their age was greater than 65 years (AOR 0.37, 95% CI 0.18-0.72; *P*<.01; Multimedia Appendix 3). Sensitivity analyses that used a 3-category dependent variable (prefer video, prefer in-person, or “about the same”) revealed few differences in predictors of preferences for video visits (Multimedia Appendix 4). An additional predictor for video visits relative to an in-person visit included age older than 65 years and a driving distance of 16 to 40 miles from the VA (relative risk ratios [RRR] 1.66, 95% CI 1.01-2.72; *P*=.04), as well as lower video visit preference among patients age ≥65 years(RRR 0.40, 95% CI 0.18-0.90; *P*=.03). The original model predictors remained significant (feeling uncomfortable in the VA, communicating concerns, number of conditions, and substance use disorder). An ordered logistic regression found similar results; significant predictors for video visits included feeling uncomfortable in the VA, communicating concerns, and substance use disorder, and patients ≥65 years were less likely to prefer video visits (AOR 0.47, 95% CI 0.27-0.82; *P*=.01; Multimedia Appendix 5).

Qualitative analyses revealed four themes underlying patient preferences for video-based vs in-person care: (1) the perceived opportunity to overcome access barriers, (2) the perception of the quality of care provided by video visits versus in-person care, (3) the feasibility of receiving necessary care by video visits versus in-person, and (4) technological issues. Exemplary quotes are presented in Textbox 1.

**Qualitative themes and representative quotes regarding patient care preferences (N=638).**

Opportunity to overcome access barriers:

- “Being handicapped & having no transportation, I have to make special arrangements for transportation & pack a lunch for my wife & myself”
- “Sometimes it’s nice to have a face to face visit with my psychologist and sometimes it’s nice not to have to drive 50 miles one way”
- “I would prefer video because it would expose me less to sick people. This benefits me a lot being a transplant recipient. And my caregiver wouldn't have to take off work to take me to the doctor.”

Perceptions of quality of care provided by video visits versus in-person care:

- “I get to see the provider just as if I came to VA in person so to me that is about the same or just as good.”
- “The care that I receive is the same in person or by video, excellent”

Feasibility of receiving necessary care by video visits versus in-person:

- “Sometimes doctors need to examine patients. I think it’s wonderful for therapy because all I need to do is talk.”
- “I prefer a video chat with mental health provider rather than the 2.5-hour commute for a short session. I like to see my medical doctor and orthopedic doctor in person. Video visits are a good way to have questions answered.”

Technological issues:

- “Need to give a class on how to use the tablet and make sure the connection & passwords are done right”
- “ept dropping video/calls; it’s no longer used because of our location”

## Discussion

### Principal Findings

This study describes the health care access barriers, experiences, and care preferences for VA patients who received VA-issued tablets for video visits. We identified several patient characteristics that may influence patients’ preferences for video visits, including certain diagnoses and number of conditions, comfort in the VA clinic, and communication style.

To our knowledge, this is the first nationally representative survey of VA tablet recipients examining their experiences with VA video visits. The strong satisfaction ratings for tablets and the fact that characteristics such as age, income, health literacy, distance from the closest VA facility, and prior technology use were not significantly associated with tablet preference suggest that engagement in video-based care is possible for many types of patients, including those who are often considered part of the *digital divide* (ie, individuals who are older, have a low income, and have greater health or disability challenges) [51]. Providing tablets to this population may help the VA engage veterans who could otherwise be left behind in technology-focused initiatives. Initiatives that encourage patients to use their own devices are growing rapidly. Distributing devices directly to patients who lack the necessary technology can increase a health system’s capacity to reach these patients.

Findings from this survey suggest opportunities to assess potential video-based care patients for specific challenges and preferences, eg, their comfort with technology and desire for in-person encounters. The finding that communication style was associated with preferences for video visits echoes other work that identified patients with certain personality traits (eg, health information seeking and socially motivated) as more comfortable with video visits [28]. The difference in the first sensitivity analyses with the reclassified outcome combining the preference for video visits and “about the same” suggests that driving distance (16-40 miles) and older age (65-101 years) are additional factors that may influence the acceptance of video care. Further research of characteristics or traits may identify additional opportunities to improve patient engagement in video visits.

Although some work has identified that patients may opt to use video care in lieu of in-person primary care [30], the nature of the program we are studying has enabled video visits to be used as an adjunct to in-person care to increase access to providers. The results indicate the importance of identifying patients who are amenable to using technology for their care and identifying opportunities to improve training for patients and providers who want to conduct video visits. Health care programs could consider patients’ chronic conditions and access barriers to identify candidates who may prefer video encounters and review the patients’ local broadband capability to ensure connectivity. Previous work has identified that patients sometimes decline telehealth owing to the lack of access or skills needed to engage in video visits [52,53] and that patients with mental health conditions are less likely to have access to the internet and technology [25,54]. Despite the technical challenges that may hinder initial use, once patients participate in a video visit, they often perceive it to be of the same or better quality than in-person care [16,17,27,29]. However, patients acknowledge that video visits cannot fully replace in-person care, particularly when physical examinations are needed for decision making [18]. This study builds on prior work by identifying additional factors such as patient communication style, comfort in the care setting, and health conditions that predict a preference for video visits when the barrier to accessing technology is removed. Telemedicine is more sensitive to patient preferences because it is the mode of health care service delivery [31] rather than a treatment option, and understanding patient preferences will enable health care systems to target this limited resource to ensure it is utilized effectively.

Some limitations of this study include a potential bias introduced by survey nonresponse, despite weighting. Even though the survey respondents were older, they were similar to the population of tablet recipients in most characteristics (Multimedia Appendix 2). Owing to the novel focus of this survey, we included some *de novo* questions, although most of the survey material was derived from validated measures [36-41]. The factor analysis of the access barriers combining big and small groupings could cause us to miss some nuances among some of the factors. However, analyses of the groupings only identified two barriers (uncomfortable or uneasy and travel time) for which the proportion differed significantly between big and small barriers by preference for care. As there were no significant differences noted for the other six factors, and because some factors had relatively small numbers in the *big problem* category, we chose to combine the big and small categories in the analyses. It is to be noted that our evaluation does not include veterans who participate in video visits from their own devices, and providers may have selectively distributed tablets to certain types of patients during this pilot, so our results may not extend to all current or potential veteran patients participating in video visits. Another limitation in interpreting our results is that we cannot attribute changes in satisfaction directly to receipt of the tablet, as surveys were only distributed to tablet recipients and there was no control group with which to compare these outcomes.

Nevertheless, results from this evaluation will inform efforts to improve the reach of this program across participating VA facilities by identifying characteristics associated with preferences for video-based care and the reasons behind these preferences. This information can help VA identify and better engage patients who may be interested in this limited resource as well as address factors that may be limiting tablet use among some populations. This study also clarifies that patients understand that video visits may not be appropriate in all cases, which can be used to inform patient and provider trainings on the appropriateness of offering video visits. For patients who prefer video visits, the VA can utilize the tablets to encourage engagement in programs and services that previously were out of reach owing to access barriers. The role that internet connectivity plays in our findings for patient preference and other research related to health care access underscores the importance of broadband access as a priority in the United States [55]. VA program offices continue to work with broadband carriers on this issue and actively test opportunities to augment this barrier by offering multiple broadband service providers or providing cellular signal boosters to patients in certain areas. The VA health care system serves an older population compared with other US health care systems [56], so the program’s success among older veterans (mean age 58.6 years among survey respondents and 54.6 years among all tablet recipients) also provides insights into how best to optimize the use of telehealth and video visits among older adults.

### Conclusions

Technology is playing an increasingly important role in enhancing health care access and delivery for patients, especially for those who are geographically isolated or homebound. Although VA has evolved to become both a provider and payer of care, its priority of ensuring access to high-quality care for veterans has not changed. The 2018 Mission Act further expands the role of telemedicine in the VA, including the approved use of video visits in the home and across state lines [57]. Critical issues remain owing to variations in broadband infrastructure that will influence the adoption and use of these technologies. This study provides important information about patient experiences with VA-issued tablets and their preferences for video vs in-person care. The findings may inform the development of assessment and training tools to improve patient targeting and support for tablet recipients as well as opportunities to improve engagement in video visits.

## Appendix

Multimedia Appendix 1 Baseline and follow-up surveys.

Multimedia Appendix 2 Weighted characteristics table.

Multimedia Appendix 3 Sensitivity analysis 1.

Multimedia Appendix 4 Sensitivity analysis 2: multinomial regression.

Multimedia Appendix 5 Sensitivity analysis 3: ordered regression.

## Abbreviations

AOR: adjusted odds ratio
DALC: Denver Acquisitions and Logistics Center
ICD: International Statistical Classification of Disease
RRR: relative risk ratios
VA: Department of Veterans Affairs
