# Supplementary material for: Patient Perceptions of Video Visits Using Veterans Affairs Telehealth Tablets: Survey Study
Source: J Med Internet Res. 2020 Apr 15;22(4):e15682. doi: 10.2196/15682 (PMC7191342; doi:10.2196/15682)
Supplement: Multimedia Appendix 1 [file jmir_v22i4e15682_app1.docx]

**Please fill out this survey as soon as you receive your tablet.**

**Evaluation of Tablet-Enabled Telehealth to Enhance Veterans’ Access and Care**


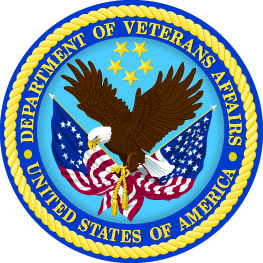


**If you misplace the enclosed pre-paid envelope for returning this survey,** please call (650) 617-2637 to request a new envelope. Alternatively, you may mail the completed survey to the following address using your own envelope. **To ensure your confidentiality**, only write personal or identifying information on this cover page, and nowhere else in the survey.

**Connected Health Study**

**795 Willow Rd. MPD-152**

**Menlo Park, CA 94025**

Please indicate who is completing this survey.

- Person who is receiving the tablet
- A family member, friend, or caregiver of the person who is receiving the tablet
- Other:

**As soon as your survey is received, we will mail you a $10 gift card to thank you for your participation. Please provide the address where you would like to receive your gift card.**

**Name: __________________________ Last 4: __________**

**Address: _______________________­­­­­__________**

**_________________________________**

**_________________________________**

*******SKIP*******

******BLANK PAGE******

**Date: _______________________**

1. **The goal of this survey is to learn about Veterans who are receiving VA care through video tablets. When did you first receive a tablet from the VA?**

- Today
- Less than one month ago
- Between 1 to 3 months ago
- Over 3 months ago

1. **Have you used the VA tablet to communicate by video with a VA clinician or staff?**

- No
- Yes, from my home or work
- Yes, from a VA clinic

1. **The next questions ask about your experience using technology to support your health.**
2. Which of the following activities have you performed using the computer, a cell phone, tablet, or other technology? (*Check all that apply*):

- Searched for health information
- Used telehealth technology to monitor my health conditions (e.g., blood pressure, blood sugar, weight) and send updates to my clinical team
- Used My Health*e*Vet (the VA’s patient portal) to renew my medications
- Used My Health*e*Vet to check my VA records (e.g., labs, test results, notes)
- Communicated with my VA health care team by secure messaging or email
- Used video (on a personal tablet or computer) to have an appointment with a VA clinician
- Communicated with other patients about a health problem (e.g., through an online support group or Facebook)
- Used a mobile phone app to manage or track a health problem

1. How frequently do you use a computer, a cell phone, or tablet to help you take care of your health and/or manage your health care?

- Daily
- 1-5 times per week
- 1-3 times per month
- Less than once per month
- Never

1. **Please indicate the degree to which you agree with the following statements.**

|  | **No experience** | **Strongly disagree** | **Disagree** | **Neutral** | **Agree** | **Strongly agree** |
| --- | --- | --- | --- | --- | --- | --- |
| Video visits could help me access my VA providers when I need them |  |  |  |  |  |  |

1. **Please describe where you typically receive your medical care.**

|  | **Mostly at**  **the VA** | **Mostly outside VA** | **About half in VA, half outside VA** | **Nowhere** |
| --- | --- | --- | --- | --- |
| Where do you receive most of your health care? |  |  |  |  |
| Where do you receive your primary care?  (e.g., care for chronic conditions, check-ups, minor illnesses) |  |  |  |  |
| Where do you receive mental health care?  (e.g., care for depression, anxiety, PTSD) |  |  |  |  |
| Where do you receive emergency care  (e.g., if you go to an emergency room)? |  |  |  |  |
| Where do you normally receive hospital care  (e.g., if you are hospitalized for medical or surgical care)? |  |  |  |  |

1. **People sometimes have problems getting health care. For each of the following items, please indicate whether it is a big problem, a small problem, or not a problem for you in getting the health care you need.**

|  | **A big problem** | **A small problem** | **Not a problem** | **Don’t know** |
| --- | --- | --- | --- | --- |
| Travel time to VA(s) where you receive care |  |  |  |  |
| Difficulty getting transportation to the VA where you receive care |  |  |  |  |
| Cost of traveling to VA(s) where you receive care  (e.g., public transportation, gas, parking) |  |  |  |  |
| Work or school make it difficult for you to get the health care you need |  |  |  |  |
| Family/caregiving responsibilities make it difficult for you to get the health care you need |  |  |  |  |
| Health conditions that make it challenging for you to get to the VA  (e.g. chronic pain, weakness, TBI, polytrauma) |  |  |  |  |
| Feeling out of place, uncomfortable, or uneasy at the VA |  |  |  |  |
| Bad weather conditions |  |  |  |  |

1. **We are interested in understanding personal costs for Veterans seeking care from the VA. Please complete the following questions about transportation to the VA clinic where you receive most of your VA care.**
2. How far away is the facility where you receive most of your VA care? miles
3. How long did it take you to get to your last appointment at this facility? minutes
4. What type of care did you receive at this facility during your last appointment?

- Primary Care
- Mental Health Care
- Specialist Care
- Other

1. How did you travel to your last appointment at this facility, and how much did it cost?

|  |  | | **Cost (Round Trip)** | |
| --- | --- | --- | --- | --- |
|  | Personal transportation (e.g., car, ride from friend) | Parking: $ | | Gas: $ |
|  | VA-provided transportation | Cost: $ | | |
|  | Public transport (e.g., bus, subway, commuter train) | Cost: $ | | |
|  | Specialized bus or van for persons with disabilities | Cost: $ | | |
|  | Other (e.g., taxi, Uber, Lyft) | Cost: $ | | |

1. How much (if any) did the VA reimburse you for this travel? $
2. If you work, how many hours did you have to take off work to attend your last appointment?

- _______ hours of paid leave
- _______ hours of unpaid leave
- I did not take time off work
- I do not currently work

1. **We would now like to hear about your general experiences with VA health care. Considering your experiences with VA health care in the last six months, how much do you agree or disagree with the following statements?**

|  | **Strongly disagree** | **Somewhat disagree** | **Neither disagree nor agree** | **Somewhat agree** | **Strongly agree** |
| --- | --- | --- | --- | --- | --- |
| I got the service I needed |  |  |  |  |  |
| It was easy to get what I needed |  |  |  |  |  |
| I got my VA care in a setting that was convenient for me |  |  |  |  |  |
| I have a VA healthcare provider who I trust |  |  |  |  |  |
| I trust VA to fulfill our country's commitment to veterans. |  |  |  |  |  |

1. **Next we would like to hear about your interactions with VA providers in the last six months.**

|  | **Never** | **Sometimes** | **Usually** | **Always** |  |
| --- | --- | --- | --- | --- | --- |
| When you needed care right away, how often did you get care as soon as you thought you needed? |  |  |  |  | **N/A** |
| How often did your providers explain things in a way that was easy to understand? |  |  |  |  |  |
| How often did your providers listen carefully to you? |  |  |  |  |  |
| How often did your providers spend enough time with you? |  |  |  |  |  |
| How often did you get the primary care that you needed? |  |  |  |  |  |
| How often did you get the mental health care you needed? |  |  |  |  |  |
| How often did you get the specialty care you needed? |  |  |  |  |  |

1. **On a scale where 1 is very satisfied and 10 is very dissatisfied, how would you rate your satisfaction with your:**

|  | **Very Very**  **Satisfied Dissatisfied** | | | | | | | | | | **I did not receive these services** |
| --- | --- | --- | --- | --- | --- | --- | --- | --- | --- | --- | --- |
|  | **1** | **2** | **3** | **4** | **5** | **6** | **7** | **8** | **9** | **10** |  |
| Overall VA Care |  |  |  |  |  |  |  |  |  |  |  |
| Primary Care (e.g., care for chronic conditions, check-ups, minor illnesses) |  |  |  |  |  |  |  |  |  |  |  |
| Mental Health Care (e.g., care for depression, anxiety, PTSD) |  |  |  |  |  |  |  |  |  |  |  |
| Spinal Cord Injury Care, if relevant |  |  |  |  |  |  |  |  |  |  |  |

1. **The next questions are about your current health and quality of life.**

|  | **Poor** | **Fair** | **Good** | **Very good** | **Excellent** |  |
| --- | --- | --- | --- | --- | --- | --- |
| In general, would you say your health is… | |  |  |  |  |  |
| In general, would you say your quality of life is… | |  |  |  |  |  |
| In general, how would you rate your mental health, including your mood and ability to think… | |  |  |  |  |  |

1. **Over the last 2 weeks, how often have you been bothered by any of the following problems?**

|  | **Not at all** | **Several days** | **More than half the days** | **Nearly every day** |
| --- | --- | --- | --- | --- |
| Little interest or pleasure in doing things |  |  |  |  |
| Feeling down, depressed or hopeless |  |  |  |  |

1. **How much are the following statements currently true for you?**

|  | **Not True** | **A Little True** | **Somewhat True** | **Mostly True** | **Very True** |
| --- | --- | --- | --- | --- | --- |
| When I see my provider I bring a list of questions or concerns I want to talk about. |  |  |  |  |  |
| I can make sure my concerns are fully addressed before my appointment ends. |  |  |  |  |  |
| When I need more information I ask, even when my provider is in a rush. |  |  |  |  |  |
| I attend all of my medical appointments. |  |  |  |  |  |
| It is easy for me to refill medications on time. |  |  |  |  |  |
| I know I can always find a way to contact my health care team. |  |  |  |  |  |

1. **Please describe your experience filling out medical forms.**

|  | **Not at all** | **A little bit** | **Somewhat** | **Quite** | **Extremely** |
| --- | --- | --- | --- | --- | --- |
| How confident are you filling out medical forms by yourself? |  |  |  |  |  |

1. **Finally, please complete the following questions about your home environment.**
2. Do you usually live with others or alone?

- Alone
- With others

1. How much do you feel you can count on having someone to:

|  | **Not at all** | **A little** | **Somewhat** | **Mostly** | **Completely** |
| --- | --- | --- | --- | --- | --- |
| Help you if you were confined to bed? |  |  |  |  |  |
| Take you to the doctor if you need it? |  |  |  |  |  |
| Prepare your meals if you are unable to do it yourself? |  |  |  |  |  |
| Help with daily chores if you were sick? |  |  |  |  |  |
| Talk about your problems or stressful events in your life? |  |  |  |  |  |

1. What is the highest grade or level of school that you have completed?

- Did not complete high school
- High school graduate or GED
- Some college or 2-year degree
- 4-year college graduate or more

1. What is your annual household income from all sources, including disability and other government support?

- Less than $25,000/year
- $25,001 - $50,000/year
- More than $50,000/year

1. Based on the household income listed above, complete the following statement.

|  | **With great difficulty** | **With difficulty** | **With some difficulty** | **Rather easily** | **Easily** | **Very easily** |
| --- | --- | --- | --- | --- | --- | --- |
| My household can make ends meet… |  |  |  |  |  |  |

**Thank you for completing this survey. Once we receive your survey we will mail you a $10 gift card to thank you for your time and contribution. We will also contact you with one more survey in 3-6 months to learn about your experience using the tablet.**

*Follow-Up Survey*

**Evaluation of Tablet-Enabled Telehealth to Enhance Veterans’ Access and Care**


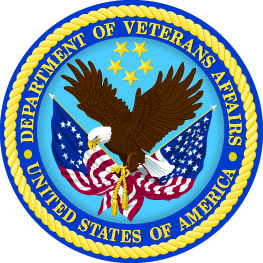


**If you misplace the enclosed pre-paid envelope for returning the survey**, please call

(650) 617-2637 to request a new envelope. Alternatively, you may mail the completed survey to the following address using your own envelope. **To ensure your confidentiality**, only write personal or identifying information on this cover page, and nowhere else in the survey.

**Connected Health Study**

**795 Willow Rd, MPD-152**

**Menlo Park, CA 94025**

Please indicate who is completing this survey.

- Person who received a VA tablet
- A family member, friend, or caregiver of the person who received a VA tablet
- Other:

**As soon as your survey is received, we will mail you a $10 check to thank you for your participation.**

**Date: _____________**

1. **First, we have some questions about your experience using technology to support your health.**
2. **How frequently do you use a computer, tablet, or mobile phone to help you take care of your health and/or manage your health care?**

- Daily
- 1-5 times per week
- 1-3 times per month
- Less than once per month
- Never

1. **Which of the following activities have you performed using the computer, a cell phone, tablet, or other technology? (*Check all that apply*):**

- Searched for health information
- Used telehealth technology to monitor my health conditions (e.g., blood pressure, blood sugar, weight) and send updates to my clinical team
- Used My Health*e*Vet (the VA’s patient portal) to renew my medications
- Used My Health*e*Vet to check my VA records (e.g., labs, test results, notes)
- Communicated with my VA health care team by secure messaging or email
- Used video (on a personal tablet or computer) to have an appointment with a VA clinician
- Communicated with other patients about a health problem (e.g., through an online support group or Facebook)
- Used a mobile phone app to manage or track a health problem

1. **The goal of this survey is to learn about care that Veterans receive through TABLETS. Our records indicate that you received a tablet from VA in the past year. Have you or someone in your home used your tablet?**

- Yes I have used the VA tablet *(****Continue to question 3****)*
- I received a tablet but I did not want to use it *(****Skip to question 7a****)*
- I received a tablet but I was unable to use it *(****Skip to question 8****)*
- I did not receive a tablet *(****Skip to question 8****)*

1. **Who is the primary person that uses the tablet?**

- Myself
- A family member or friend
- Other (e.g., home aide, visiting nurse, caregiver)

1. **Please tell us about your experience with the tablet equipment and technology.**

|  | **Does Not Apply** | **Strongly Disagree** | **Disagree** | **Neutral** | **Agree** | **Strongly Agree** |
| --- | --- | --- | --- | --- | --- | --- |
| I was satisfied with the time it took for the tablet to arrive |  |  |  |  |  |  |
| I got the help I needed to learn how to use the tablet |  |  |  |  |  |  |
| The tablet equipment is easy to use |  |  |  |  |  |  |
| I can always trust the tablet to work |  |  |  |  |  |  |
| I feel the tablet technology is secure and that my information is kept confidential |  |  |  |  |  |  |
| When using the tablet, I can hear my provider clearly and feel that they can hear me |  |  |  |  |  |  |
| When using the tablet, I can see my provider clearly and feel they can see me |  |  |  |  |  |  |
| I have experienced technical difficulties during video calls |  |  |  |  |  |  |
| I have experienced technical difficulties sending vital sign information using the tablet equipment (e.g. blood pressure, weight, pulse) |  |  |  |  |  |  |
| There is enough technical support to help me complete my video visit(s) |  |  |  |  |  |  |

**5. We would now like to hear about your experience using the tablet for VA care.**

|  | **Does Not Apply** | **Strongly Disagree** | **Disagree** | **Neutral** | **Agree** | **Strongly Agree** |
| --- | --- | --- | --- | --- | --- | --- |
| It is easy for me to ask my doctor questions during video visits |  |  |  |  |  |  |
| It is easy for me to ask for help if I don’t understand something during video visits |  |  |  |  |  |  |
| It is easy for me to understand my doctor’s instructions during video visits |  |  |  |  |  |  |
| My healthcare provider can adequately address my health concerns during video visits |  |  |  |  |  |  |
| The lack of physical contact during a video visit is not a problem |  |  |  |  |  |  |

**6. Please describe your experience using the tablets to access VA care.**

|  | | **Does Not Apply** | **Strongly Disagree** | **Disagree** | **Neutral** | **Agree** | | **Strongly Agree** | |
| --- | --- | --- | --- | --- | --- | --- | --- | --- | --- |
| Video visits help me access my VA providers when I need them | |  |  |  |  |  | |  | |
| Video visits help me get care that I couldn’t access otherwise | |  |  |  |  |  | |  | |
| Video visits with VA providers save me time | |  |  |  |  |  | |  | |
| Video visits help me involve family and friends in my care | |  |  |  |  |  | |  | |
| My health is better than it was before I started using the tablet | |  |  |  |  |  | |  | |
| Overall I am satisfied with my video visit(s) | |  |  |  |  |  | |  | |
| I would recommend video visit(s) to other veterans | |  |  |  |  |  | |  | |
| Video visits with VA providers save me money (e.g., cost of transportation, gas, lodging, food) | |  |  |  |  |  | |  | |
| If you agreed with the above statement, approximately how much money do you save per appointment? | - Less than $25 - Between $25 and $50 - Between $50 and $75 - Between $75 and $100 - More than $100 | | | | | |  |  |  |
| Video visits help me avoid missing work to attend VA appointments | |  |  |  |  |  | |  | |
| If you agreed with the above statement, approximately how much leave do you typically take to attend a VA appointment? | - Less than 3 hours **paid** leave - 3-5 hours **paid** leave - More than 5 hours **paid** leave - Less than 3 hours **unpaid** leave - 3-5 hours **unpaid** leave - More than 5 hours **unpaid** leave - I usually do not take leave to attend appointments | | | | | | | |  |

**7a. Please consider the healthcare you’ve received since receiving your tablet.**

|  | **Prefer Video** | **Prefer**  **In-Person** | **About the Same** | **Does Not Apply** |
| --- | --- | --- | --- | --- |
| Overall, how do you prefer to get your healthcare from the VA? |  |  |  |  |

**7b. Please explain the preference you indicated in question 7a.**

**__________________________________________________________________________**

**__________________________________________________________________________**

**__________________________________________________________________________**

**__________________________________________________________________________**

**8. Please consider all your recent experiences with the VA since you’ve received your tablet. How much do you agree or disagree with the following statements?**

|  | **Strongly Disagree** | **Somewhat Disagree** | **Neither Disagree Nor Agree** | **Somewhat Agree** | **Strongly Agree** |
| --- | --- | --- | --- | --- | --- |
| I got the service I needed |  |  |  |  |  |
| It was easy to get what I needed |  |  |  |  |  |
| I got my VA care in a setting that was convenient for me |  |  |  |  |  |
| I have a VA healthcare provider who I trust |  |  |  |  |  |
| I trust VA to fulfill our country's commitment to veterans. |  |  |  |  |  |

**9. On a scale where 1 is very dissatisfied and 10 is very satisfied, how would you rate your satisfaction with your:**

|  | **Very Very**  **Dissatisfied Satisfied** | | | | | | | | | | **I did not receive these services** |
| --- | --- | --- | --- | --- | --- | --- | --- | --- | --- | --- | --- |
|  | **1** | **2** | **3** | **4** | **5** | **6** | **7** | **8** | **9** | **10** |  |
| Overall VA Care |  |  |  |  |  |  |  |  |  |  |  |
| Primary Care (e.g., care for chronic conditions, check-ups, minor illnesses) |  |  |  |  |  |  |  |  |  |  |  |
| Mental Health Care (e.g., care for depression, anxiety, PTSD) |  |  |  |  |  |  |  |  |  |  |  |
| Care for Alcohol or Drug Problems |  |  |  |  |  |  |  |  |  |  |  |
| Spinal Cord Injury Care  (e.g., care for conditions related to spinal cord injury) |  |  |  |  |  |  |  |  |  |  |  |

**10. Are you ready to return your tablet to the VA? Yes No**

**11. When you are ready to return your tablet, how do you plan to return it to the VA?**

- Give it to my provider
- Give it to a telehealth coordinator at my VA facility
- Request a tablet retrieval kit and mail it in a pre-paid box
- Not sure

**12. Please add any additional comments about your experience with the VA Tablet program:**

**_____________________________________________________________________________**

**_____________________________________________________________________________**

**_____________________________________________________________________________**

**Thank you for participating in this evaluation. We will send you a $10 check when we receive this survey to thank you for your time.**
