# Supplementary material for: Patient Perceptions of Video Visits Using Veterans Affairs Telehealth Tablets: Survey Study
Source: J Med Internet Res. 2020 Apr 15;22(4):e15682. doi: 10.2196/15682 (PMC7191342; doi:10.2196/15682)
Supplement: Multimedia Appendix 2 [file jmir_v22i4e15682_app2.docx]

**Appendix Table B. Post Stratification Weighting Characteristics**

|  | Unweighted Baseline and Follow-up Respondents (N=764) | | | | Weighted Baseline and Follow-up Respondents (N=764) | | | Tablet Cohort (N=5,981) | | | |
| --- | --- | --- | --- | --- | --- | --- | --- | --- | --- | --- | --- |
|  | N | % | Mean (SD) | Median (IQR) | N | % | Mean (SE) | N | % | Mean (SD) | Median (IQR) |
| **Age (years)** |  |  | 58.7 (14.4) | 60.5 (49-69.2) |  |  | 55.6 (0.2) |  |  | 55.4 (17.0) | 56.8 (39.9-68.6) |
| **Age Categories** |  |  |  |  |  |  |  |  |  |  |  |
| 18-44 | 142 | 18.6 |  |  | 137 | 29.9 |  | 1,865 | 31.2 |  |  |
| 45-64 | 323 | 42.3 |  |  | 306 | 35.0 |  | 2,038 | 34.1 |  |  |
| 65-101 | 299 | 39.1 |  |  | 299 | 35.2 |  | 2,078 | 34.7 |  |  |
| **% Male** | 624 | 81.7 |  |  | 624 | 79.4 |  | 5,031 | 84.1 |  |  |
| **Marital Status** |  |  |  |  |  |  |  |  |  |  |  |
| Married | 452 | 59.2 |  |  | 450 | 57.9 |  | 3,149 | 52.9 |  |  |
| Divorced/never married | 286 | 37.5 |  |  | 287 | 40.2 |  | 2,517 | 42.3 |  |  |
| Widowed | 25 | 3.3 |  |  | 17 | 1.9 |  | 288 | 4.8 |  |  |
| **Race** |  |  |  |  |  |  |  |  |  |  |  |
| White or White non-Hispanic | 604 | 80.3 |  |  | 598 | 80.3 |  | 4,559 | 78.4 |  |  |
| Black or African American | 91 | 12.1 |  |  | 90 | 12.3 |  | 704 | 12.1 |  |  |
| American Indian, Native Hawaiian, or other | 18 | 2.4 |  |  | 18 | 2.3 |  | 205 | 3.5 |  |  |
| Asian | 4 | 0.5 |  |  | 4 | 0.8 |  | 59 | 1.0 |  |  |
| Unknown or decline | 35 | 4.7 |  |  | 34 | 4.4 |  | 288 | 5.0 |  |  |
| **Ethnicity** |  |  |  |  |  |  |  |  |  |  |  |
| Hispanic or Latino | 32 | 4.2 |  |  | 32 | 5 |  | 307 | 5.2 |  |  |
| Not Hispanic/Latino | 706 | 92.7 |  |  | 704 | 92.1 |  | 5,443 | 92.0 |  |  |
| Unknown or decline | 24 | 3.1 |  |  | 24 | 3 |  | 165 | 2.8 |  |  |
| **Rurality^b^** |  |  |  |  |  |  |  |  |  |  |  |
| Urban | 341 | 45.3 |  |  | 342 | 46.2 |  | 2,745 | 47.5 |  |  |
| Rural or highly rural | 411 | 54.7 |  |  | 412 | 53.8 |  | 3,037 | 52.5 |  |  |
| **Education^a^** |  |  |  |  |  |  |  |  |  |  |  |
| HS attended or graduate/GED | 227 | 29.8 |  |  | 227 | 28.5 |  |  |  |  |  |
| Some college or 2-year degree | 343 | 45.1 |  |  | 343 | 45.6 |  |  |  |  |  |
| 4-year college graduate or more | 191 | 25.1 |  |  | 191 | 25.8 |  |  |  |  |  |
| Income ^a^ |  |  |  |  |  |  |  |  |  |  |  |
| < $25,000/year | 249 | 33.6 |  |  | 249 | 33 |  |  |  |  |  |
| $25,001-$50,000/year | 324 | 43.7 |  |  | 324 | 44 |  |  |  |  |  |
| > $50,000/year | 169 | 22.8 |  |  | 169 | 23.1 |  |  |  |  |  |
| **Economic Hardship^a^** |  |  |  |  |  |  |  |  |  |  |  |
| Great Difficulty/Difficulty | 243 | 32.1 |  |  | 243 | 32.8 |  |  |  |  |  |
| Some difficulty | 271 | 35.8 |  |  | 271 | 35.4 |  |  |  |  |  |
| Rather easily | 149 | 19.7 |  |  | 149 | 19.2 |  |  |  |  |  |
| Easily or Very Easily | 94 | 12.4 |  |  | 94 | 12.6 |  |  |  |  |  |
| **Driving Distance to Primary Facility (Miles)** | 756 |  | 22.9 (22.9) | 16 (7-32) | 761 |  | 22.8 (0.8) | 5,875 |  | 22.6 (22.3) | 16  (6-32) |
| **Health Literacy (out of 4)^a^** | 755 |  | 2.6 (1.3) | 3 (2-4) | 755 |  | 2.6 (0.1) |  |  |  |  |
| **Technology Use pre-tablet^a^**  **(out of 8)** | 761 |  | 2.6 (1.9) | 2 (1-4) | 761 |  | 2.7 (0.1) |  |  |  |  |
| **% 4G Internet coverage** | 760 |  | 96.5 (10.5) | 99.99 (99.1-100) | 760 |  | 96.6  (.35) | 5,891 |  | 97.0 (10.3) | 99.9 (99.4-100) |
| **Tablet Use at 6 months** | 592 |  | 5.7 (5.2) | 3 (1-6) | 567 |  | 5.5 (0.21) | 592 |  | 5.7 (5.2) | 4 (2-7) |
| **Mental Health Tablet Appointments at 6 months** | 383 |  | 5.7 (5.0) | 4 (2-8) | 368 |  | 5.1 (0.25) | 383 |  | 5.7 (5.0) | 4 (2-8) |
| **Total Number of Conditions** |  |  | 4.1 (2.3) | 4 (2-5) |  |  | 3.89 (0.07) |  |  | 4.1 (2.4) | 4 (2-6) |
| **Mental Health Conditions** | 561 | 73.1 | 1.2 (1.1) | 1 (0-2) |  | 73.0 | 1.3 (0.03) | 4,367 | 73.0 | 1.3 (1.1) | 1 (0-2) |
| **Number of Conditions** |  |  |  |  |  |  |  |  |  |  |  |
| 0-3 | 297 | 38.9 |  |  | 297 | 41.1 |  | 2,487 | 41.6 |  |  |
| 4 -6 | 339 | 44.4 |  |  | 339 | 43.8 |  | 2,266 | 37.9 |  |  |
| 7 -14 | 128 | 16.8 |  |  | 128 | 15.1 |  | 1,228 | 20.5 |  |  |
