# Supplementary material for: Patient Perceptions of Video Visits Using Veterans Affairs Telehealth Tablets: Survey Study
Source: J Med Internet Res. 2020 Apr 15;22(4):e15682. doi: 10.2196/15682 (PMC7191342; doi:10.2196/15682)
Supplement: Multimedia Appendix 3 [file jmir_v22i4e15682_app3.docx]

**Appendix C.** **Characteristics associated with preference for either video visits and video/in-person visits “about the same”**

| Prefer video appointments or “about the same” | Adjusted Odds Ratio^a^ | P-value | [95% Conf. Interval] | |
| --- | --- | --- | --- | --- |
| VA Technology use^b^ | 1.22 | 0.38 | 0.78 | 1.91 |
| Other Technology use^b^ | 1.13 | 0.54 | 0.77 | 1.65 |
| Reliance on VA: Medical Care^c^ | 0.82 | 0.44 | 0.50 | 1.36 |
| Reliance on VA: Mental Health Care^c^ | 1.04 | 0.88 | 0.62 | 1.74 |
| Drive Distance to Assign VA (ref: <15miles) |  |  |  |  |
| 16-40 miles | 1.65 | **0.02** | 1.09 | 2.51 |
| >40 miles | 1.33 | 0.34 | 0.74 | 2.38 |
| Access Barriers: Transport/travel^d^ | 1.66 | 0.08 | 0.94 | 2.91 |
| Access Barriers: Commitments^d^ | 1.13 | 0.57 | 0.74 | 1.72 |
| Access Barrier: Uncomfortable/Uneasy^d^ | 1.21 | 0.40 | 0.77 | 1.89 |
| Gender (ref: Male) | 1.02 | 0.93 | 0.61 | 1.71 |
| Age Categories (ref: 18-44) | |  |  |  |
| 45-64 | 0.62 | 0.11 | 0.34 | 1.12 |
| 65-101 | 0.37 | **0.00** | 0.18 | 0.72 |
| Married^a^ | 0.73 | 0.12 | 0.49 | 1.08 |
| Verizon coverage (ref: less than 95% coverage) | 1.52 | 0.17 | 0.84 | 2.75 |
| Economic Hardship (Some/great difficulty making ends meet v all else) | 1.26 | 0.32 | 0.80 | 1.98 |
| Education (ref: Some college or more) |  |  |  |  |
| High school graduate/GED | 1.26 | 0.33 | 0.80 | 1.98 |
| When I see my provider I bring a list of questions or concerns I want to talk about^c^ | 0.95 | 0.82 | 0.64 | 1.43 |
| I can make sure my concerns are fully addressed before my appointment ends^c^ | 1.39 | 0.12 | 0.92 | 2.11 |
| Health Literacy (Quite/Extremely v all else) | 1.02 | 0.91 | 0.68 | 1.53 |
| Total # Conditions (continuous) | 0.95 | 0.32 | 0.87 | 1.05 |
| Any SUD^b^ | 1.72 | 0.06 | 0.97 | 3.04 |
| Depression^b^ | 1.00 | 0.99 | 0.65 | 1.54 |
| PTSD^b^ | 1.36 | 0.15 | 0.90 | 2.06 |
| Schizophrenia/Bipolar^b^ | 0.71 | 0.40 | 0.32 | 1.58 |
| Constant | 0.99 | 0.99 | 0.29 | 3.41 |
| N | 558 |  |  |  |

^a^ Multivariate logistic regression comparing the characteristics of patients who reported preferring video visits or reported preferring video and in-person care as “about the same” to those who reported preferring in-person care (reference group); ^b^Any or yes v none; ^c^Mostly/True v all else; ^d^Big or small problem v Not a problem/don’t know; PTSD: Post Traumatic Stress Disorder; SUD: Substance Use Diagnoses
