# Supplementary material for: Patient Perceptions of Video Visits Using Veterans Affairs Telehealth Tablets: Survey Study
Source: J Med Internet Res. 2020 Apr 15;22(4):e15682. doi: 10.2196/15682 (PMC7191342; doi:10.2196/15682)
Supplement: Multimedia Appendix 4 [file jmir_v22i4e15682_app4.docx]

**Appendix D. Multinomial Regression**

|  | RRR | P>\|t\| | [95% Conf. Interval] | |
| --- | --- | --- | --- | --- |
| **Video** |  |  |  |  |
| VA Technology use^a^ | 1.45 | 0.18 | 0.84 | 2.50 |
| Other Technology use^a^ | 1.03 | 0.91 | 0.64 | 1.65 |
| Reliance on VA: Medical Care^b^ | 0.82 | 0.51 | 0.47 | 1.46 |
| Reliance on VA: Mental Health Care^b^ | 0.81 | 0.49 | 0.45 | 1.46 |
| Drive Distance to Assign VA (ref: <15miles) |  |  |  |  |
| 16-40 miles | 1.66 | **0.04** | 1.01 | 2.72 |
| >40 miles | 1.68 | 0.13 | 0.85 | 3.32 |
| Access Barriers: Transport/travel^c^ | 1.80 | 0.11 | 0.87 | 3.71 |
| Access Barriers: Commitments^c^ | 1.17 | 0.54 | 0.71 | 1.93 |
| Access Barrier: Uncomfortable/Uneasy^c^ | 1.96 | **0.01** | 1.17 | 3.27 |
| Gender (ref: Male) | 0.97 | 0.93 | 0.53 | 1.77 |
| Age Categories (ref: 18-44) |  |  |  |  |
| 45-64 | 0.70 | 0.31 | 0.36 | 1.38 |
| 65-101 | 0.40 | **0.03** | 0.18 | 0.90 |
| Married^a^ | 0.98 | 0.92 | 0.61 | 1.57 |
| Verizon coverage (ref: less than 95% coverage) | 1.90 | 0.09 | 0.90 | 4.02 |
| Economic Hardship (Some/great difficulty making ends meet v all else) | 1.52 | 0.11 | 0.90 | 2.56 |
| Education (ref: Some college or more) |  |  |  |  |
| High school graduate/GED | 1.55 | 0.11 | 0.90 | 2.65 |
| When I see my provider I bring a list of questions or concerns I want to talk about^b^ | 1.27 | 0.34 | 0.78 | 2.08 |
| I can make sure my concerns are fully addressed before my appointment ends^b^ | 1.75 | **0.03** | 1.05 | 2.94 |
| Health Literacy (Quite/Extremely v all else) | 1.06 | 0.80 | 0.65 | 1.73 |
| Total # Conditions (continuous) | 0.88 | **0.05** | 0.77 | 1.00 |
| Any SUD^a^ | 2.34 | **0.01** | 1.20 | 4.53 |
| Depression^a^ | 0.98 | 0.94 | 0.59 | 1.63 |
| PTSD^a^ | 1.50 | 0.11 | 0.92 | 2.46 |
| Schizophrenia/Bipolar^a^ | 1.01 | 0.99 | 0.41 | 2.46 |
| **In person (base outcome)** |  |  |  |  |
| **About the same** |  |  |  |  |
| VA Technology use^a^ | 1.06 | 0.81 | 0.65 | 1.75 |
| Other Technology use^a^ | 1.21 | 0.38 | 0.78 | 1.88 |
| Reliance on VA: Medical Care^b^ | 0.83 | 0.50 | 0.48 | 1.44 |
| Reliance on VA: Mental Health Care^b^ | 1.32 | 0.35 | 0.73 | 2.38 |
| Drive Distance to Assign VA (ref: <15miles) |  |  |  |  |
| 16-40 miles | 1.64 | **0.04** | 1.03 | 2.61 |
| >40 miles | 1.11 | 0.75 | 0.58 | 2.14 |
| Access Barriers: Transport/travel^c^ | 1.55 | 0.17 | 0.83 | 2.88 |
| Access Barriers: Commitments^c^ | 1.12 | 0.62 | 0.71 | 1.77 |
| Access Barrier: Uncomfortable/Uneasy^c^ | 0.81 | 0.43 | 0.48 | 1.36 |
| Gender (ref: Male) | 1.08 | 0.78 | 0.61 | 1.91 |
| Age Categories (ref: 18-44) |  |  |  |  |
| 45-64 | 0.58 | 0.09 | 0.31 | 1.09 |
| 65-101 | 0.35 | **0.01** | 0.17 | 0.74 |
| Married^a^ | 0.58 | **0.02** | 0.37 | 0.91 |
| Verizon coverage (ref: less than 95% coverage) | 1.33 | 0.39 | 0.70 | 2.53 |
| Economic Hardship (Some/great difficulty making ends meet v all else) | 1.09 | 0.73 | 0.66 | 1.81 |
| Education (ref: Some college or more) |  |  |  |  |
| High school graduate/GED | 1.05 | 0.84 | 0.63 | 1.76 |
| When I see my provider I bring a list of questions or concerns I want to talk about^b^ | 0.76 | 0.22 | 0.49 | 1.18 |
| I can make sure my concerns are fully addressed before my appointment ends^b^ | 1.18 | 0.47 | 0.75 | 1.87 |
| Health Literacy (Quite/Extremely v all else) | 0.99 | 0.97 | 0.63 | 1.56 |
| Total # Conditions (continuous) | 1.01 | 0.82 | 0.92 | 1.12 |
| Any SUD^a^ | 1.37 | 0.33 | 0.73 | 2.55 |
| Depression^a^ | 1.01 | 0.96 | 0.62 | 1.65 |
| PTSD^a^ | 1.26 | 0.32 | 0.80 | 2.00 |
| Schizophrenia/Bipolar^a^ | 0.50 | 0.14 | 0.20 | 1.26 |
| N | 558 |  |  |  |

^a^Any or yes v none; ^b^Mostly/True v all else; ^c^Big or small problem v Not a problem/don’t know; PTSD: Post Traumatic Stress Disorder; SUD: Substance Use Diagnoses
