# Supplementary material for: Patient Perceptions of Video Visits Using Veterans Affairs Telehealth Tablets: Survey Study
Source: J Med Internet Res. 2020 Apr 15;22(4):e15682. doi: 10.2196/15682 (PMC7191342; doi:10.2196/15682)
Supplement: Multimedia Appendix 5 [file jmir_v22i4e15682_app5.docx]

**Appendix E. Ordered Regression**

| Prefer Video Appointments  (0: in person, 1: same, 2: video) | Adjusted Odds Ratio | P>\|t\| | [95% Conf. Interval] | |
| --- | --- | --- | --- | --- |
| VA Technology use^a^ | 1.28 | 0.21 | 0.87 | 1.89 |
| Other Technology use^a^ | 1.02 | 0.91 | 0.72 | 1.44 |
| Reliance on VA: Medical Care^b^ | 0.85 | 0.41 | 0.57 | 1.26 |
| Reliance on VA: Mental Health Care^b^ | 0.81 | 0.36 | 0.52 | 1.27 |
| Drive Distance to Assign VA (ref: <15miles) |  |  |  |  |
| 16-40 miles | 1.39 | 0.06 | 0.99 | 1.96 |
| >40 miles | 1.49 | 0.14 | 0.88 | 2.52 |
| Access Barriers: Transport/travel^c^ | 1.53 | 0.11 | 0.91 | 2.55 |
| Access Barriers: Commitments^c^ | 1.06 | 0.75 | 0.74 | 1.53 |
| Access Barrier: Uncomfortable/Uneasy^c^ | 1.67 | **0.01** | 1.17 | 2.39 |
| Gender (ref: Male) | 0.95 | 0.79 | 0.64 | 1.41 |
| Age Categories (ref: 18-44) |  |  |  |  |
| 45-64 | 0.80 | 0.31 | 0.52 | 1.23 |
| 65-101 | 0.47 | **0.01** | 0.27 | 0.82 |
| Married^a^ | 0.98 | 0.90 | 0.71 | 1.36 |
| Verizon coverage (ref: less than 95% coverage) | 1.48 | 0.12 | 0.90 | 2.43 |
| Economic Hardship (Some/great difficulty making ends meet v all else) | 1.34 | 0.12 | 0.93 | 1.93 |
| Education (ref: Some college or more) |  |  |  |  |
| High school graduate/GED | 1.41 | 0.10 | 0.94 | 2.10 |
| When I see my provider I bring a list of questions or concerns I want to talk about^b^ | 1.23 | 0.25 | 0.86 | 1.77 |
| I can make sure my concerns are fully addressed before my appointment ends^b^ | 1.44 | **0.04** | 1.02 | 2.04 |
| Health Literacy (Quite/Extremely v all else) | 1.05 | 0.81 | 0.73 | 1.49 |
| Total # Conditions (continuous) | 0.93 | 0.10 | 0.85 | 1.01 |
| Any SUD^a^ | 1.71 | **0.02** | 1.11 | 2.66 |
| Depression^a^ | 0.97 | 0.88 | 0.69 | 1.38 |
| PTSD^a^ | 1.35 | 0.09 | 0.95 | 1.91 |
| Schizophrenia/Bipolar^a^ | 1.07 | 0.86 | 0.51 | 2.27 |
| n | 558 |  |  |  |

^a^Any or yes v none; ^b^Mostly/True v all else; ^c^Big or small problem v Not a problem/don’t know; PTSD: Post Traumatic Stress Disorder; SUD: Substance Use Diagnoses
